# The role of artificial intelligence in transforming maternity services in Africa: prospects and challenges

**DOI:** 10.1177/26334941241288587

**Published:** 2024-10-15

**Authors:** Obasanjo Afolabi Bolarinwa, Aliu Mohammed, Victor Igharo

**Affiliations:** Department of Public Health, York St John University, 1 Clove Street, London, E14 2BA, UK; Demography and Population Studies Programme, Schools of Public Health and Social Sciences, University of the Witwatersrand, Johannesburg, South Africa; Department of Health, Physical Education and Recreation, University of Cape Coast, Cape Coast, Ghana; The Challenge Initiative, Nigeria Hub, Johns Hopkins Center for Communication Programs, Abuja, Nigeria

**Keywords:** Artificial intelligence, Africa, challenges, maternity services, prospects

## Introduction

Maternal and neonatal health outcomes in Africa remain a significant public health challenge.^1^ Despite ongoing efforts to enhance maternity services, many women across the continent do not receive the necessary antenatal care (ANC), and a substantial number of births and deliveries still occur outside health facilities, leading to preventable complications and fatalities.^2^ In the same vein, postnatal care (PNC), which is also crucial for the health and well-being of mothers and their babies, is also underutilised.^2^

This trajectory continues because Africa faces hurdles, such as limited healthcare infrastructure, shortages of skilled healthcare providers, and socio-cultural barriers that impede access to maternity care.^3^ Consequently, these challenges contribute to delayed or inadequate care, exacerbating adverse maternal and neonatal health outcomes.^4^ Furthermore, the high prevalence of infectious diseases complicates pregnancy and childbirth, increasing risks for both mothers and infants.^5^

Given these pressing issues, this commentary discusses the potential of leveraging artificial intelligence (AI) to bridge the gaps in maternity care, such as ANC, health facility deliveries and PNC across the continent. AI could offer substantial promise in addressing these gaps in maternity services delivery and utilisation, with various applications that could detect maternal health issues such as pregnancy-related complications or preterm labour.^6^ Similarly, AI can also predict post-delivery complications.^7^ By leveraging AI, there is a significant opportunity to enhance maternal and newborn health outcomes in Africa, ensuring comprehensive and timely care throughout the maternity continuum.

## The importance of maternity services in Africa

According to the World Health Organisation (WHO),^1^ countries in Africa, particularly those in sub-Saharan Africa (SSA), account for approximately two-thirds of maternal deaths worldwide. Additionally, nearly two-fifths of global stillbirths^8^ and neonatal deaths^9^ occur on the continent. Consequently, maternal and neonatal health outcomes in Africa remain significantly high compared to average global prevalence. Although various interventions have been implemented to scale up maternity services provision and utilisation to improve maternal and child outcomes in Africa, however, recent estimates revealed that only 59% of pregnant women in some African countries within SSA receive the recommended four or more ANC visits.^2^ Besides, a significant number of deliveries still occur outside health facilities,^10^ often leading to preventable complications and maternal and child deaths.

Furthermore, PNC, which is crucial for the health and well-being of postpartum women and their babies, remains hugely underutilised, with only about 50% of mothers accessing the service.^11^ AI offers significant potential to improve maternal and newborn health outcomes in Africa by addressing gaps in maternity services. Its applications could help to detect and stop early maternal health complications relating to gestational diabetes with over 90% accuracy or preterm labour, leading to timely intervention^6^ and, more importantly, predict and prevent post-delivery issues.^7^

## Enhancing antenatal care with artificial intelligence

Promoting positive ANC experience is essential in increasing the number of ANC visits, ensuring compliance with ANC-related interventions, minimising the risk of pregnancy-related complications, and ensuring early detection and management of complications.^6^ Through predictive analytics using pregnant clients’ data (e.g., maternal health records and socio-demographic factors), AI algorithms can analyse large datasets to predict ANC defaulters and women at risk of pregnancy-related complications,^6^ allowing healthcare providers to institute targeted interventions to reduce ANC default rate and address high-risk pregnancies. For instance, AI technology can predict women at risk of gestational diabetes during the ANC period,^7^ enhancing both clients’ and care provider awareness, which could guide interventions such as clients’ education and thereby minimise the risk and potential complications of the condition. Besides, AI has proven to be beneficial in the management of gestational diabetes among ANC attendants, with a high rate of clients’ compliance with blood glucose monitoring.^7^ For instance, A study in Nigeria demonstrated that AI-driven systems reduced ANC defaulter rates by 15% by using predictive analytics to identify women at risk of complications and sending alerts for follow-up visits.^12^

Also, AI-driven chatbots and virtual health assistants can provide round-the-clock support, reminding expectant mothers of their ANC appointments and offering health education tailored to the needs of pregnant women,^6^ thus enhancing ANC outcomes.

## Improving health facility deliveries

Although health facility deliveries in Africa have increased over the past few decades, home deliveries remain relatively high in many countries,^7^ contributing to the high prevalence of maternal and perinatal mortality on the continent. Predictive AI models can increase health facility deliveries by providing real-time data on labour onset,^6^ enabling expectant mothers to seek delivery care at health facilities on time and thereby minimising delivery-related complications and mortality. AI solutions for optimising resource allocation have been shown to reduce maternal complications by 30% in low-resource settings.^13^ Also, AI applications can optimise resource allocation and improve emergency response times during deliveries. For instance, AI algorithms can prompt healthcare providers on labour onset and forecast demand for maternity services, including the need for caesarean sections, helping facilities prepare adequately with necessary supplies and skilled personnel.^14^ Moreover, AI-powered telemedicine platforms can bridge the gap between remote areas and skilled healthcare professionals, providing real-time consultations and decision support during labour and delivery.^14^ Further, AI models can detect premature uterine contractions^15^ or monitor foetal heart rate to predict the risk of complications during labour,^16^ enabling healthcare providers to intervene on time and thereby prevent premature deliveries and other labour-related complications.

## Streamlining postnatal care

PNC is often neglected in many low-middle income countries, particularly in Africa, despite being essential for the health of mothers and their newborn babies. Evidence suggests that only half of postpartum women in African countries in SSA utilise PNC services.^11^ The use of AI applications in PNC could provide an invaluable opportunity to improve access and utilisation of PNC by 19% through appointment reminders and symptom tracking.^17^ For example, wearable devices equipped with AI technology can alert mothers of their next PNC visits and track vital health indicators, alerting clinicians to post-delivery-related health risks such as postpartum haemorrhages^18^ and depression,^19^ ensuring timely interventions to mitigate these risks. Also, AI techniques built on multiple sets of baby images and weights can be used to estimate the gestational age of babies during PNC, allowing care providers to determine prematurity and provide age-appropriate interventions.^20^ Additionally, AI-driven mobile applications such as a virtual assistant can guide new mothers on PNC practices, including nutrition and breastfeeding, and respond to pertinent PNC issues,^20^ thus promoting easy access to PNC health information and guides on how to address pertinent postnatal health problems.

**Figure 1.**
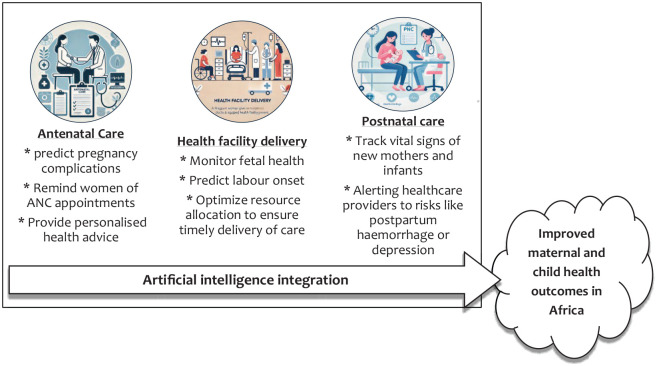
Conceptualising AI integration into maternity services in Africa. AI, artificial intelligence.

Figure 1 presents a conceptual framework for integrating AI into maternity services in Africa, showcasing how technology can transform key areas of maternal and child care. In ANC, AI tools can predict pregnancy complications, provide timely reminders for appointments, and offer personalised health advice, helping to ensure that mothers receive the care they need. For Health Facility Deliveries, AI can play a crucial role in predicting labour onset, monitoring foetal health, and optimising resources to guarantee timely and safe deliveries. PNC benefits from AI-enabled wearables and systems that track the health of both mother and child, sending alerts to healthcare providers about potential risks like postpartum haemorrhage or depression. By weaving AI into the fabric of maternity services, this framework promises to drive improved maternal and child health outcomes across Africa, making care smarter, more responsive, and ultimately safer.

## Overcoming infrastructure and technological barriers

Whilst integration of AI into maternity services has the potential to improve maternal and child outcomes in Africa, infrastructural and technological limitations remain a major hindrance towards such innovation. For instance, in spite of the significant improvement in telecommunication technology and expertise over the years, many people in Africa still do not have access to internet services and smart devices, including smartphones and computers, which are required to run most AI applications.^21^ The high cost of data and AI-supportive devices could also curtail the consistent use of AI in maternity services in Africa. Besides, the nonavailability of reliable sources of power, such as electricity, in many parts of Africa could affect the use of AI technologies in maternity services, especially in rural communities. Therefore, to realise the benefits of integrating AI into maternity services, concerted efforts and collaborations are required from key stakeholders, including governments, telecommunication providers, healthcare providers, and AI developers, to ensure the availability of the needed technological infrastructure and reliable internet connectivity, since investments in digital infrastructure and capacity building are necessary steps towards creating an enabling environment for AI technologies.^22^ For instance, governments can collaborate with internet service providers to ensure that AI applications integrated into maternity services are zero-rated to warrant consistency of use. Also, developers of AI applications must consider the limited technological skills of clients and clinicians in Africa when developing AI applications, ensuring that the applications are user-friendly to enhance utilisation. Since AI applications are data-driven, it is important to digitise maternal health records and maternal data generation in healthcare facilities in Africa. This could promote effective data training and enhance the precision of AI applications in maternity services.

## Building a skilled workforce

The adoption of AI in healthcare requires a skilled workforce capable of operating AI applications and maintaining AI systems. Therefore, training healthcare professionals in AI literacy and technical skills is crucial for the effective integration of AI in maternity services in Africa. This could be achieved through partnerships with healthcare providers, educational institutions and AI developers. Healthcare institutions, AI developers and international organisations can facilitate the development of training programmes to build the skills and capacity of healthcare workers and ensure local expertise in AI applications.^22^ Considering that errors in data generation could lead to false predictions and misapplied interventions,^23^ there is a need to enhance the skills and capacity of maternity care providers in Africa in data handling to avoid aggregated errors in data generation.

## Addressing data privacy and security concerns

Whilst data quality and completeness are essential in developing effective AI algorithms, the need to ensure that client data remains private and secure cannot be overemphasised. The implementation of AI in healthcare raises significant concerns about data privacy and security. For instance, the sensitivity of health data necessitates the need for robust measures to protect client information from authorised access and misuse.^22^ Thus, to gain public trust and safeguard clients’ privacy in the utilisation of AI in maternity services, it is important to establish stringent data protection regulations, including data access and management protocols, and ensure compliance.^22^

## Navigating ethical and cultural considerations

While acknowledging the significance of AI in improving maternity services, it is important to consider the inherent ethical and cultural issues to promote acceptance and utilisation. For instance, because individuals develop AI algorithms, potential biases in algorithm generation may occur,^21^ which could lead to mistrust and cause resistance to the use of AI technology in maternity services. Also, bias in the training of data sets is a major concern in advancing the use of AI in healthcare in Africa since most available AI tools were developed based on data from clients in advanced countries, which could affect their predictive abilities among clients in Africa.^23^ Thus, engaging healthcare providers, local communities and other stakeholders in the development and implementation process of AI applications in maternity services could help address these concerns. It is also important to ensure that AI applications are culturally appropriate and equitable, providing benefits across diverse maternal populations, including women with disabilities.^23^ Also, there is a need for regulations to guide the use of AI and prevent the application of experimental AI in the provision of maternity services.

## Conclusion

Considering the complexity of pregnancies and the risk of developing complications before, during and after childbirth, AI holds a substantial promise in improving maternity services in Africa by enhancing ANC provision, improving health facility deliveries, and streamlining PNC. However, realising these potentials requires addressing significant challenges, including removing infrastructural and technological barriers, enhancing the technological skills of healthcare providers, safeguarding data quality and data privacy, and ensuring ethical and culturally appropriate use of AI technologies in maternity services. With concerted efforts from governments, international organisations and the private sector, AI can become a powerful tool in advancing maternity services across the continent and thereby improving maternal and perinatal outcomes in Africa.

## Appendix

### Abbreviations

ANC Antenatal care

PNC Postnatal care

AI Artificial Intelligence

WHO World Health Organisation

SSA Sub-Saharan Africa

LMICs Low-middle income countries.

## Declarations

**Ethics approval and consent to participate:** Not applicable.

**Consent for publication:** Not applicable.

**Availability of data and materials:** Not applicable.
